# Dynamic Behaviors of Condensing Clusters Based on Rayleigh Scattering Experiment

**DOI:** 10.1038/s41598-017-01190-9

**Published:** 2017-04-20

**Authors:** Zhong Lan, Di Wang, Kejian Cao, Quan Xue, Xuehu Ma

**Affiliations:** 1 State Key Laboratory of Fine Chemicals, Liaoning Key Laboratory of Clean Utilization of Chemical Resources, Institute of Chemical Engineering, Dalian University of Technology, Dalian, 116024 China; 2 NO. 6 Academy of CASIC, Huhhot, 010076 China

## Abstract

Condensation is a common physical process which widely exists in natural phenomena and thermal energy systems. In a condensation process, cluster is considered as the important bridge between vapor body and condensates. However, limited by the minimum imaging dimension of traditional measurements, early experimental studies about initial stages of condensation process are not sufficient. This paper provides a powerful optical platform for the study of dynamic clusters process. Based on the Rayleigh law, optical experiments were firstly introduced to investigate the clusters spatial distribution close to and far from condensation surface. The results show that clusters are mainly generated in the vicinity of the condensation surface within the thickness of 200 *μ*m. When they move away from the condensation surface, clusters progressively vanish and they have a life cycle of a fraction of a millisecond. Though scattering intensity is proportional to the 6th power of cluster radius *r* and cluster number density *N*_c_ theoretically, the scattering intensity does not increase sharply with the increase of subcooling degree from the experimental results, so we can infer that the cluster number density plays a dominate role in this process and the effect of cluster radius almost can be ignored.Zhong Lan and Di Wang contributed equally to this work.

## Introduction

Condensation and its heat transfer commonly exist in natural phenomena and industrial applications^1–5^. In the process of dropwise condensation, single droplet undergoes a whole process of nucleation, growth, coalescence and departure^6,7^. Nucleation has received widespread attention because it occurs not only in the initial stage of dropwise condensation but also filmwise condensation^8–10^. Although the processes after nucleation are well understood, water molecule clusters before nucleation, which are considered as original state of condensation, are relatively unexplored. In order to reveal the microphysical processes from vapor to condensates, cluster physical model was proposed to explain the initial stage of condensation in early studies^11–13^. It is presumed that steam molecules become clusters with the lognormal size distribution in bulk steam phase. However, it lacked the mechanism of formation and distribution of cluster itself because it just investigated the process of cluster sedimentation and the model has not been confirmed. In addition, molecular simulations were also applied to demonstrate cluster phenomena because of their abilities to observe the physical processes of nucleation which usually occur within nanometer spatial scale and nanosecond time scale^14–17^. However, it cannot demonstrate spatial changes of clusters near cooling surface. Theories about cluster physical model and methods of molecular simulations provide qualitative descriptions and quantitative explanations about vapor droplet microcosmic views. From the previous research, whether clusters exist or not lacks direct observation and proof. The distribution of clusters influenced by the surface condition or the subcooling condition has not been confirmed. If we describe the condensation process as lifecycle, the research of nucleation process is roughly equivalent to the study of embryonic period, so it is critical to be investigated and it may lead to the new enhancement technology. Nevertheless, it is difficult to observe the physical process of nucleation stage directly through traditional testing methods due to the microscopic scale limitation.

Optical measurement methods, such as infrared spectrum^18–20^, reflection spectrum^21,22^, were applied to measure some properties of clusters. Infrared spectrum was reported on precisely size-selected clusters, with molecule number *n* ranging from 85 to 475 in bulk steam phase^23^, but it was not used in the detection of dynamic condensation process. Optical theories, like scattering^24,25^, diffraction and interference^24^, could provide effective foundations in investigating nucleation processes, which have been widely adopted for the research of simple systems such as natural gas clusters and simple water system. The size of cluster was considered as several nanometers to a few tens nanometers, which is much smaller than visible laser wavelength range 390 nm to 780 nm. The particle scattering in this magnitude follows the principal of Rayleigh law. Thus, the Rayleigh scattering can be used in the measurement of average cluster size and cluster density^26^. The advantages of Rayleigh scattering are easy to experiment and hard to destroy the size and the distribution of clusters, so Rayleigh scattering is a good method to digest the behavior of clusters. Based on the cluster formation and distribution characteristics, we try to use the Rayleigh scattering method to demonstrate cluster phenomena.

According to the Rayleigh law, the scattering of single particle follows^26^:

1 $$\frac{d\sigma }{d{\rm{\Omega }}}={\gamma }^{2}{k}^{4}$$

where σ is cross-sectional area of particle Rayleigh scattering, *γ* is polarizability of scattering particle and *k* is incident laser wavenumber,

2 $$\gamma ={r}^{3}\frac{\varepsilon -1}{\varepsilon +2}$$

where *r* is the mean radius of clusters, *ε* is permittivity of particles. For water clusters, *ε* = *n* ^2^, where *n* is the refractive index of water, *n* = 1.33. For a laser beam propagating from *x* to *x* + Δ*x* in an ensemble of clusters, the energy scattered by the clusters into a collecting lens is given by

3 $${I}_{{\rm{lens}}}={I}_{0}{\sigma }_{{\rm{lens}}}{N}_{c}{\rm{\Delta }}x$$

where *I*_lens_ is the scattering intensity probe received, *I*_0_ is the laser energy incident on the scattering volume, *σ*_lens_ is the cluster size averaged distribution cross section for Rayleigh scattering into the collecting lens, and *N*_c_ is the average number of clusters per unit volume, the spatial length is equal to ${\rm{\Delta }}x=2(h\,\cdot \,\tan \,0.22+R)$. With ${\sigma }_{{\rm{lens}}}={\int }_{lens}\frac{d\sigma }{d{\rm{\Omega }}}d{\rm{\Omega }}=\pi {k}^{4}{\gamma }^{2}({\alpha }^{2}-\frac{{\alpha }^{4}}{4})$ for 90° scattering and *kr* ≪ 1, $\alpha ={\tan }^{-1}(\frac{R}{h})\approx \frac{R}{h}$ is the scattering collection half-angle of the lens, *R*_0_ is the lens radius, and *h* is the distance between the scattering volume and lens, so we obtain

4 $${r}^{6}{N}_{c}={(\frac{{n}^{2}+2}{{n}^{2}-1})}^{2}{(\frac{\lambda }{2\pi })}^{4}\frac{1}{\pi ({\alpha }^{2}-\frac{{\alpha }^{4}}{2}){\rm{\Delta }}x}\frac{{I}_{lens}}{{I}_{0}}$$

where the $\frac{\lambda }{2\pi }$ is the laser wave number. From this equation, the *I*_lens_ is proportional to the 6th power of the mean radius of clusters *r* and average number of clusters per unit volume *N*_c_, that is:

5 $${I}_{lens}\propto {N}_{c}\cdot {r}^{6}$$

Therefore, the energy of Rayleigh scattering by clusters represents the production of clusters and it is the combined effect of cluster size and density. From previous research, the minimum radius of drops can be considered as^27,28^,

6 $${r}_{\min }=\frac{2{T}_{{\rm{v}}}{\sigma }_{{\rm{lv}}}}{{H}_{{\rm{fg}}}{\rho }_{{\rm{l}}}{\rm{\Delta }}T}$$

where *T*_v_ is the vapor temperature, *σ*_lv_ the surface tension, *H*_fg_ the latent heat of vaporization, *ρ*_1_ the density of the condensate and Δ*T* the subcooling degree. We can estimate the minimum radius of drops ranges from 2 nm to 20 nm in the condition of subcooling degree from 1 K to 10 K. Rayleigh scattering is the elastic scattering of light by particles which are much smaller than the wavelength of the radiation. In this experiment, the green laser wavelength λ is 532 nm and the size of clusters can be considered as seen Å to several nanometers. Thus, clusters can follow Rayleigh law.

From the previous research about clusters, qualitative description accounted for most part, but experimental studies are relatives less. In view of this situation, we designed relevant experiment to explore cluster behaviors and observe cluster phenomena more detailed. On the basis of Rayleigh law above, we constructed optical experimental platform to explore the behaviors of clusters. In this paper, we conducted two experiments: the experiment of cluster condensation close to surface and the cluster jet flow scattering. Clusters show different distributions in the vertical direction since jet flow causes clusters to move away from surface, so we can study the life cycle of clusters and investigate properties of cluster far from surface. The main purpose of this paper is to use optical methods to investigate the behaviors of clusters close to condensation surface and phenomena far from condensation surface based on the clusters jet flow scattering.

## Results

### Behaviors of cluster close to condensation surface

**Figure 1:**
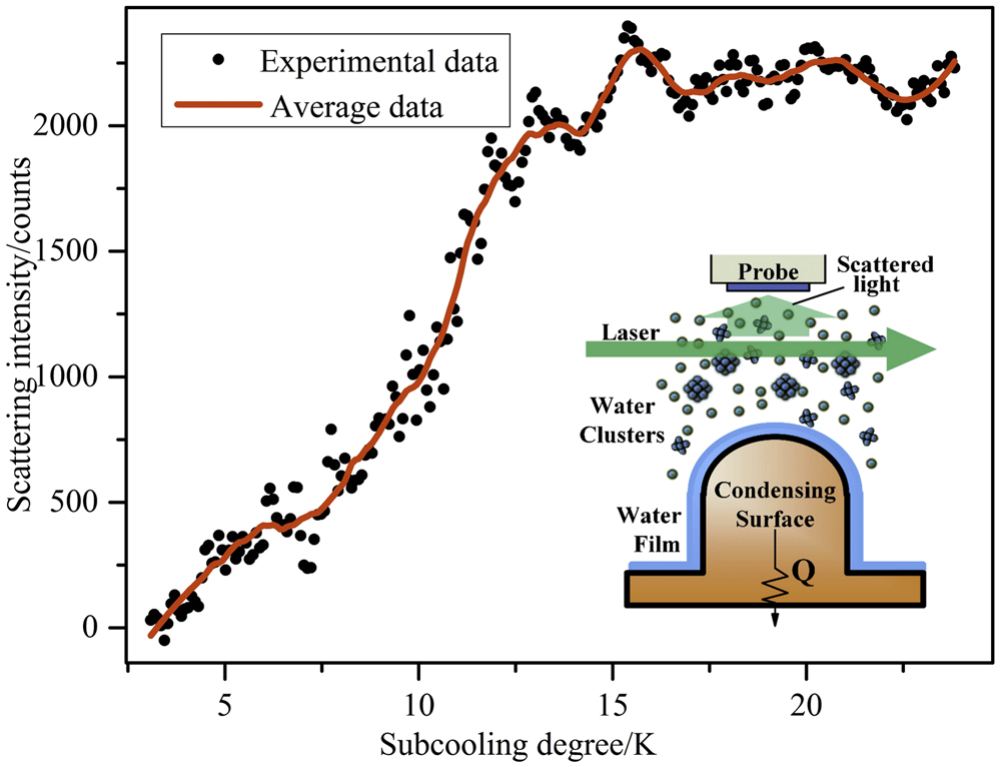
Clusters scattering intensity changes with subcooling degree.

The scattering intensity close to condensate surface in different subcooling degreess is demonstrated in Fig. 1. We designed the copper as hemisphere because it can eliminate the roughness effect caused by flat surface. Also, the diameter of fiber probe is only about 200 *μ*m, which is much smaller than the arc of the copper hemisphere with 10 mm diameter, so the hemisphere shape would not influence the detection of optical signal. From this figure, it can be found that the scattering intensity close to surface grows with the increase of subcooling degree. Scattering intensity is related to the size and density of clusters, so it can be seen that the production of vapor clusters increases with the increase of subcooling degree. Here, we need to explain that we set the scattering intensity at subcooling degree of 6 K as start point because we could guarantee that the condensation surface was completely covered by the liquid film in this experiment, and then the influence of surface irregularity can be eliminated. Therefore, the scattering intensity in Fig. 1 is relative value because of that. From curve tendency, we can see that when the subcooling degree is less than 14 K, scattering intensity approximately follows a linear relationship with the subcooling degree. The goodness of fit of this linear fitting equation is 0.934, so this curve has high fitting degree when the subcooling degree less than 14 K. However, the scattering intensity begins to level off after subcooling degree of 14 K. It can be speculated that clusters reach the number limitation and they cannot exist as vapor phase, so the scattering intensity reaches the maximum and does not change. Due to the appropriate linear relationship, we can infer that the increase of scattering intensity mainly results from *N*_c_. If mean radius *r* plays a leading rule, the scattering intensity will increase sharply and the relation between scattering intensity and subcooling degree is similar to exponential distribution. Thus, we can almost ignore the influence of mean radius *r*. From our early investigation^29^, Gibbs free energy of cluster formation Δ*G* exhibits a maximum value when a cluster containing *i* molecules achieves the critical size. And the addition of another molecule causes a decrease in Δ*G*, that is, the cluster reaches a lower and more stable energy level. Furthermore, with the increase of subcooling degree, the cluster would probably grow to a macroscopic size and phase change or condensation will happen. In this experiment, we found that, with the increase of surface subcooling degree, the curve gradually increases in an appropriately linear tendency, which demonstrates that the effect of average radius of clusters can be ignored. It also elucidates that more initial droplets break through the critical size and continue to grow up to clusters and the final sizes of clusters are almost close to each other.

**Figure 2:**
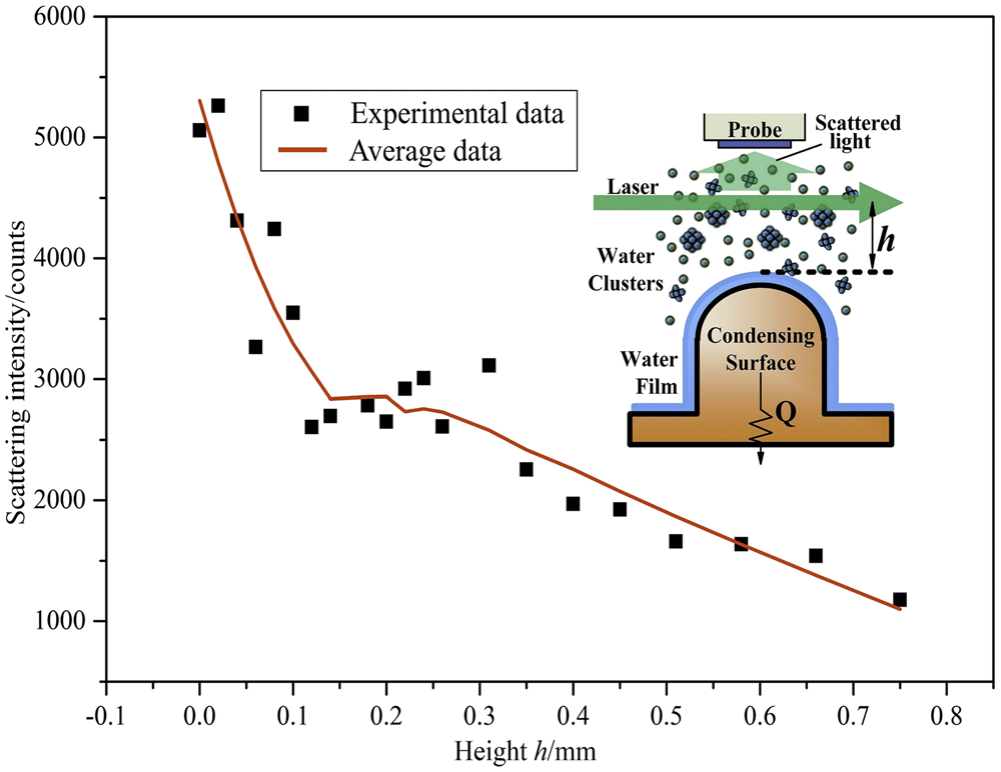
Clusters scattering intensity at different heights (Δ*T* = 20 K).

The scattering intensity at the subcooling degree of 20 K in different heights is demonstrated in Fig. 2. Here, we chose 20 K as the degree of subcooling degree in this experiment because it can make scattering intensity in a large range so the data can be analyzed more clearly. From the Fig. 2, the scattering intensity decreases as the height increases. Thus, the production of vapor clusters in vertical direction decrease when the distance between probe and surface becomes farther. At the same time, we can see that the scattering intensity falls to 50% of the initial value at a height of 200 *μ*m above the surface, falls to 20% at 700 *μ*m. It can be inferred that vapor clusters mainly distribute in the area of hundreds of micrometers far from the surface, which provides a guide in the enhancement of surface heat transfer.

### Phenomena of clusters far from condensation surface based on jet flow scattering

**Figure 3:**
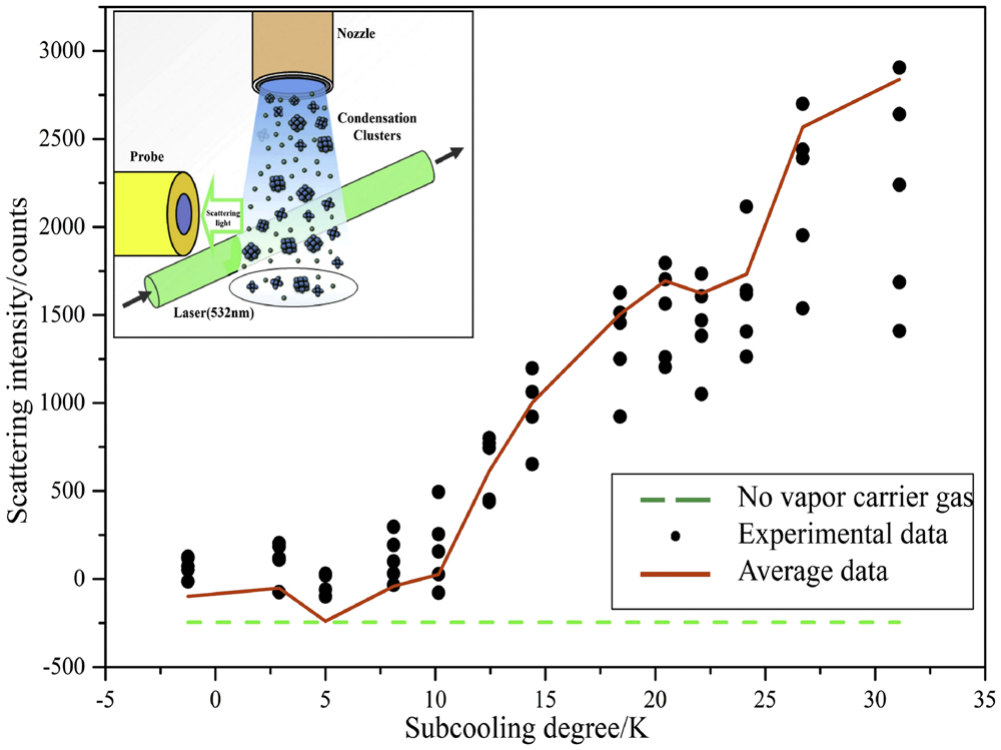
Clusters jet scattering intensity affected by subcooling degree.

From the last experiment, we can see clusters mainly exist in the vicinity of the condensation surface within the thickness of hundreds of micrometers. Clusters vanish when they move away from the surface. In order to investigate the phenomena of clusters far from the condensation surface, we designed the clusters flow scattering experiment to investigate the life cycle of clusters and got properties of dynamic clusters in that region. Make the rate of carrier gas at 19 m/s and keep constant, the fraction of steam in the nitrogen carrier is 5% and the scattering intensity in different subcooling degrees is demonstrated in Fig. 3. The diagram shows that the intensity of scattering signals increases with the enhancement of subcooling degree. Nevertheless, when the subcooling degree is less than 10 K, the scattering intensity approaches to the initial intensity. It can be deduced that there are few clusters form in relatively small subcooling degrees. Also, we can see that the production of clusters grows more rapidly when the subcooling degree becomes larger. The result is in accordance with the above results. For the carrier gas flow without vapor, temperature cannot influence scattering signals. Thus, the influence of carrier gas to scattering signals can be excluded.

**Figure 4:**
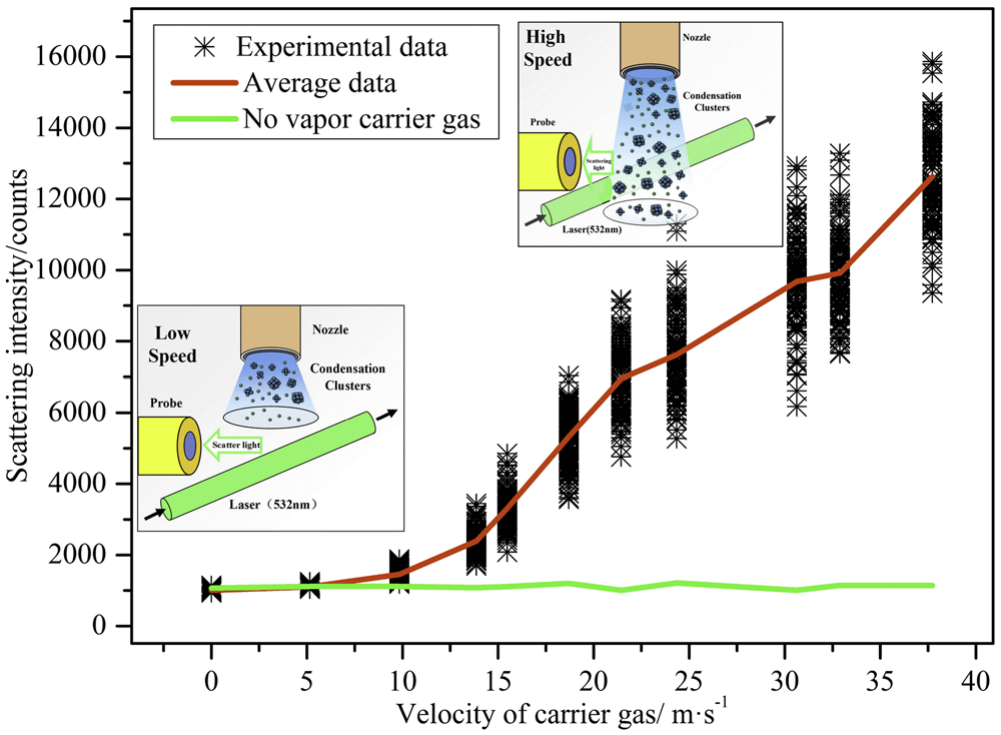
Clusters jet scattering intensity affected by carrier gas flux (Δ*T* = 15 K).

The clusters jet flow scattering intensity in subcooling degree of 15 K at different rates is demonstrated in Fig. 4. The subcooling degree 15 K is an appropriate degree since the scattering intensity will fluctuate extremely when the degree is larger. When the carrier gas rate below 10 m/s, there is no scattering signals. However, when the rate reaches a certain value larger than 10 m/s, the cluster jet flow scatters and signals can be detected by probe. In addition, the higher rate of carrier gas, the larger scattering intensity. When the mean value of scattering light intensity increases, the fluctuation of scattering signals becomes more severe. We can speculate that there are more collisions and combinations among clusters because of more kinetic energy. If there is not vapor in the carrier gas, the scattering intensity keeps in the initial value, so the pure nitrogen does not interfere scattering signals and the whole scattering signals are produced by clusters. By analysis, we can conclude that clusters have certain life span. When clusters jet flow emitted from copper pipe, clusters far away from low temperature surface begin to disappear. If the flow velocity of carrier gas is slow, clusters cannot reach the area that laser illuminates, which makes scattering signals weak. However, if the rate is high enough, clusters can get to the area and the scattering intensity is strong. Take the flow velocity of 19 m/s as example, single cluster takes about 0.1 ms from the nozzle orifice to the area that laser illuminates, so it can be concluded that the life span of clusters near the surface is the order of millisecond at the subcooling degree of 15 K.

## Discussion

Based on Rayleigh law, we observed cluster phenomena near condensate surface through optical experiment, which is unexplored in the vapor condensation process. We also analyzed the relationship between surface subcooling degree and cluster intensity. Consequently, we draw the following conclusions.

During pure steam condensation process, water molecule clusters are generated in the vicinity of condensation surface. With the increase of the subcooling degree, scattering intensity approximately follows a linear distribution. *N*_c_ plays a dominate role in this process and the effect of *r* can be ignored. In the subcooling degree larger than 14 K, clusters reach saturation. The increase of subcooling degree will lead to thickening of the cluster layer; molecular clusters mainly distributed near surface layer within several hundred micrometers thickness.

In the cluster jet flow experiment, the production of clusters grows more in the condition of subcooling degree increasing. Clusters have a life span of a fraction of a millisecond. Molecular clusters vanish when they move away from the condensation surface; the longer distance away from the surface, the greater proportion of cluster vanishes.

By the above experiments, the process of dynamic clusters before nucleation has an overall impact on the subsequent condensation process.

## Methods

### Experimental Setup of cluster condensation near surface

**Figure 5:**
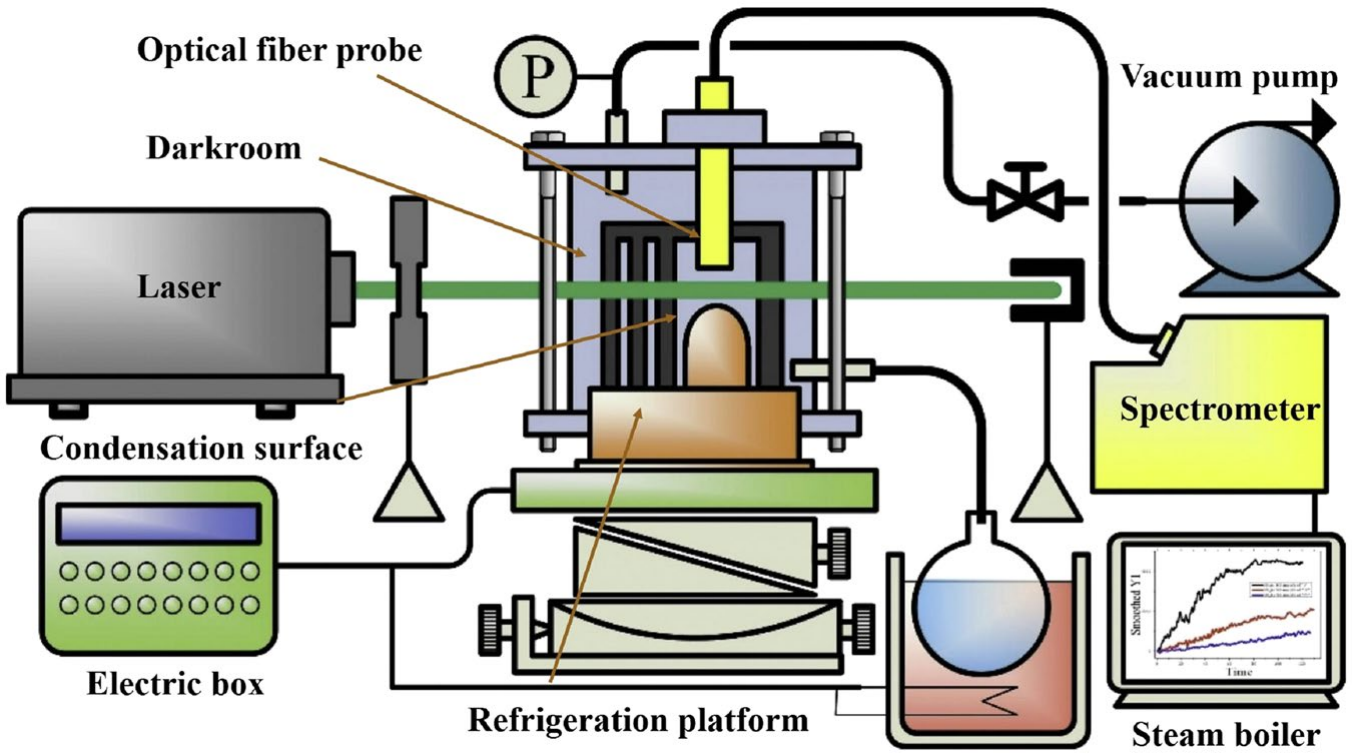
Scheme of cluster condensation near surface.

**Figure 6:**
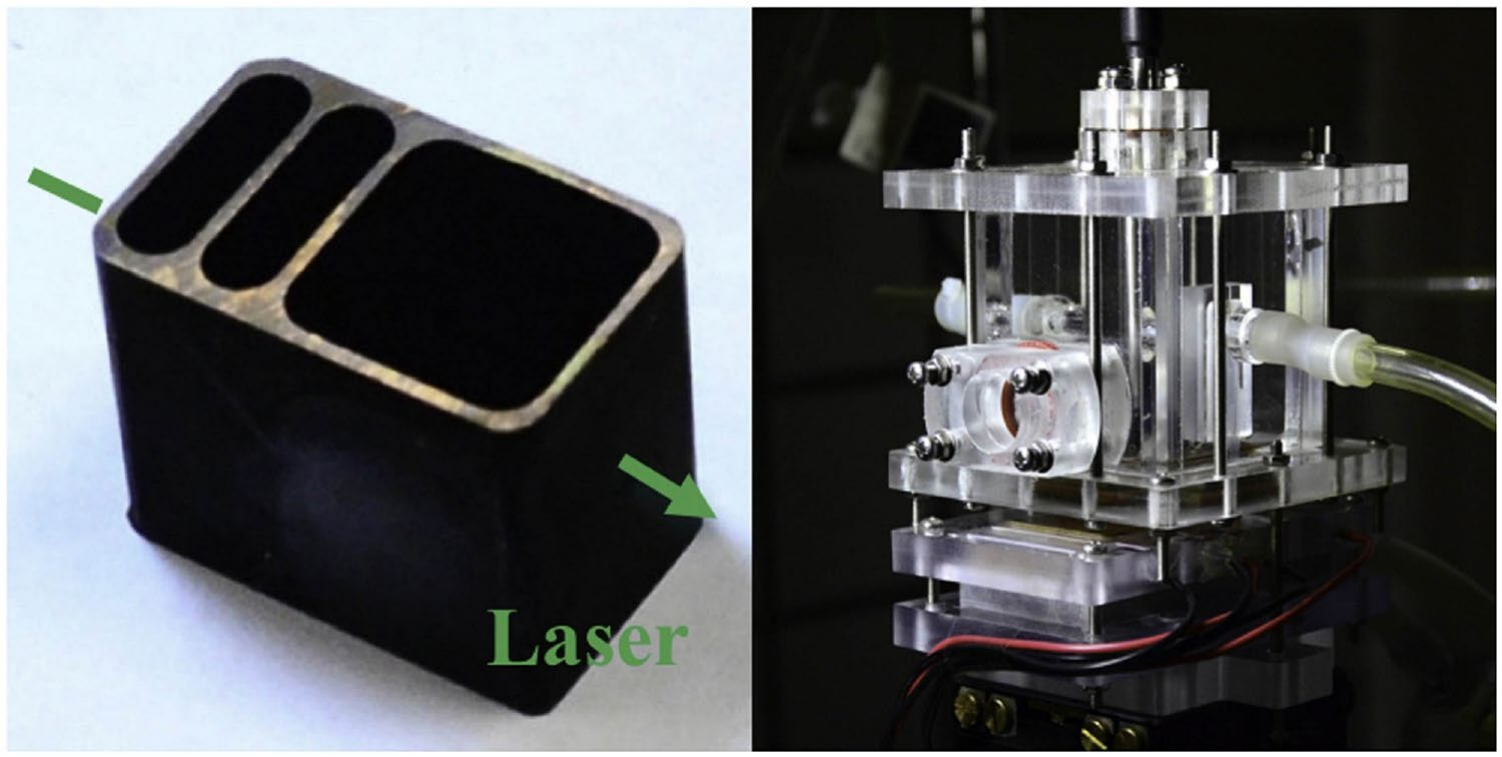
Pictures of experimental equipment.

Figure 5 shows the scheme of cluster condensation near surface. This experimental system consists of a laser source, a chamber of vacuum condensation, an optical inspection system, a temperature control system, an optical system (maintain the laser beam, lens and probe in a straight line), a vacuum pump and a steam boiler. The laser wavelength λ is 532 nm. The vacuum pump was connected with the chamber of vacuum condensation, which had good tightness to avoid leaking. A piece of copper was inlaid in the surface of the chamber to exchange heat between inside and outside. The side wall of the chamber was equipped with a round silica glass window, which made laser transmit freely. In order to eliminate interference of stray light, a darkroom was set in the chamber. In this experiment, eliminating spray light is an important issue, so we designed the darkroom as a metal hollow chamber and left a laser light path in the side wall. In order to split the rim of laser beam, three coaxial rasters were setup at the entrance of incident laser. The copper darkroom was treated with oxidized etching, so it could eliminate the spray light as much as possible and enhance signal-to-noise ratio, as shown in Fig. 6(a). A piece of hemispherical copper was placed in the darkroom, when the laser was transmitted closely to the copper surface, the vapor body was irradiated by the laser, as shown in Fig. 6(b). Hemisphere can eliminate the roughness effect caused by flat surface. The optical fiber probe was dipped into the chamber to receive the scattering signals, then the spectrometer turned scattering signals into digital signals. The steam boiler connected with chamber through pipes was used to supply steam. The temperature control system adjusted both the temperature of semiconductor chilling plate and water bath in steam boiler. The subcooling degree can be measured through a thermocouple inlaid in the copper surface. The accuracy of temperature measurement can be regulated within 0.1 K and the heat flux is less than 1000 W/m^2^. So, uncertainties of surface subcooling can be ignored. Semiconductor chilling plate could achieve the function of heating and cooling because of the Peltier effect. To regulate subcooling degree more precisely, semiconductor chilling plate fitted closely together to the bottom of copper. The chamber combined with the semiconductor chilling plate was fixed in the five dimensional optical platform, which can control x, y, z direction and two angles of dip and depression.

### Experimental Setup of cluster jet flow scattering

**Figure 7:**
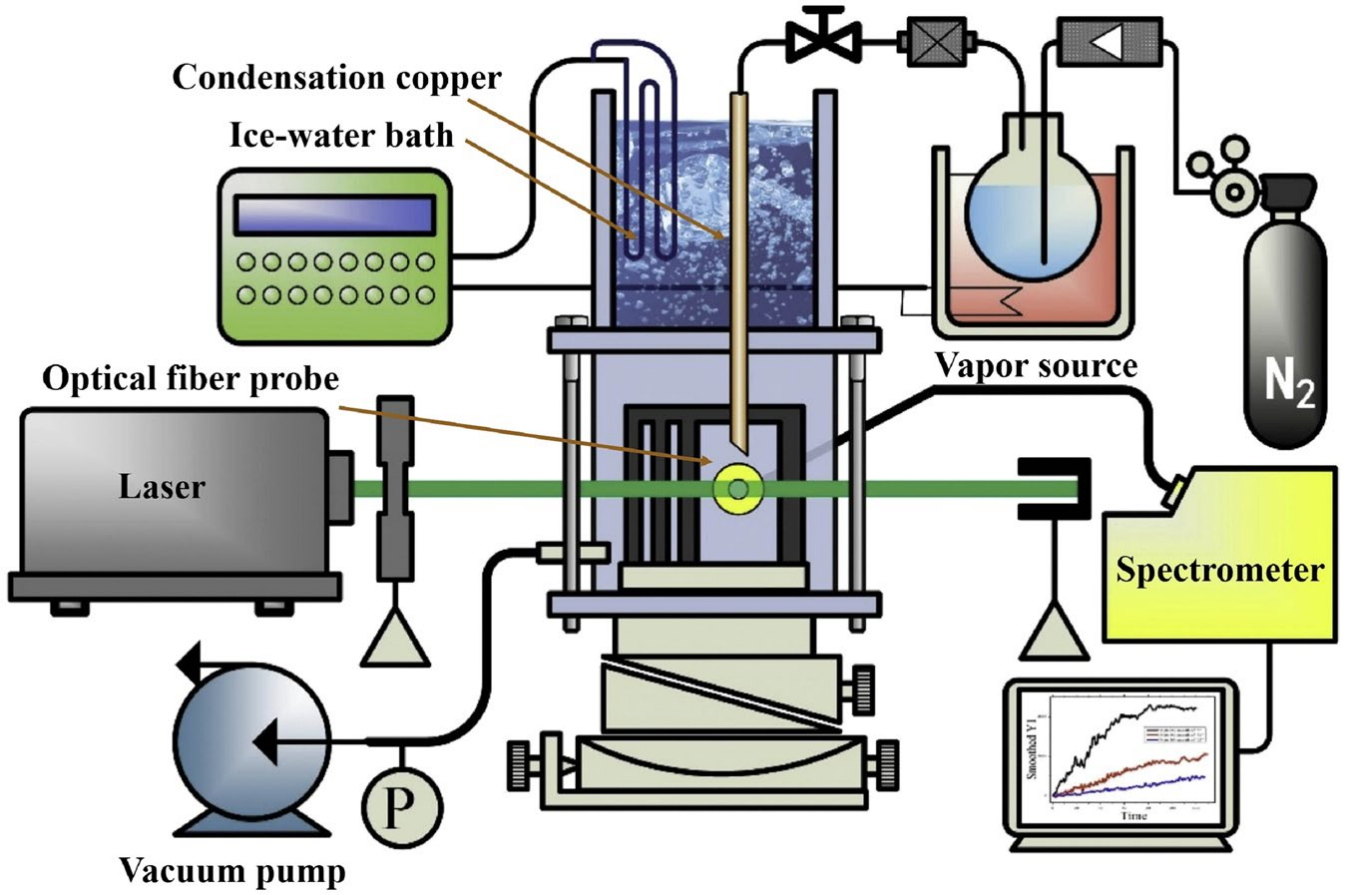
Scheme of clusters jet flow scattering.

As shown in Fig. 7, the device body of cluster jet flow scattering experiment is similar to the last experiment. High purity nitrogen as carrier gas transports vapor to the vacuum condensation chamber through steam boiler. In this experiment, a copper pipe was inserted in the ice-water bath, the temperature control system controlled the temperatures of ice-water bath and steam boiler. The vapor could condensate into supersaturated vapor in the transportation process when the temperature of ice water bath fell below water saturation temperature. Supersaturated vapor was injected into the chamber and formed cluster jet flow. When the laser beam transmitted to cluster jet flow and scattered, the probe could detect scattering signals and then the spectrometer turned scattering light signals into digital signals.

### The measurement of cluster condensation close to surface

In this measurement, the scattering intensity in different surface temperatures were measured. The detailed method is as follow: The Z-axis micrometer was adjusted to make the laser beam transmit cling to the condensation surface. We set the receiving scattering intensity at this moment as relative value *I*_R0_. Then the surface temperature decreased in a constant rate from 14 °C to −4.5 °C, the scattering intensity was recorded by the spectrometer. In order to guarantee accuracy, the measurement was repeated for 3 times.

On the basis of the last experiment, we lowered the platform to different heights. The surface temperature decreased in a constant rate from 14 °C to −4.5 °C, the scattering intensity was recorded by the spectrometer. In order to guarantee accuracy, the measurement was repeated for 3 times. Then we chose the data in different heights but at same temperature to compare their intensities.

### The measurement of cluster jet flow scattering

We set the distance between optical fiber probe and laser beam as 2 mm, and the distance between the exit of copper pipe and laser beam was 3 mm. The temperature of ice-water bath was set as 5 °C and the vapor boiler as 4 °C which ensures that the initial vapor would not condensate in ice-water bath. Other sections of this experiment were same to the last experiment.

Turn the vacuum pump and close the valve *V*_1_ of vapor boiler to extract gas and dust in the chamber, then open the valve *V*_1_, the nitrogen was added into the chamber to clean the remain gas and dust, then recorded the scattering light intensity *I*_0_ and set it as background value. In this experiment, pressure reducing valve was adjusted to a constant carrier gas flow rate at 62.5 L/h. At this carrier gas flow rate, the scattering intensity was recorded as *I*_1_. Then, the temperature of vapor boiler was increased 2.5 °C, when the temperature became stable, repeated the process above and recorded the data as *I*_2_ and so on, to the subcooling degree reached to 30 K.

The influence of carrier gas flow rate to scattering intensity of cluster jet flow was also studied. In this experiment, the temperature of ice water bath was set as 5 °C and kept constant. However, the temperature of vapor boiler was set as 30 °C. Turn the vacuum pump and close the valve *V*_1_ of vapor boiler to extract the gas and dust in the chamber, then open the valve *V*_1_, the nitrogen was added into the chamber to clean the remain gas and dust, then recorded the scattering light intensity *I*_0_ and set it as background value. Adjusted the valve *V*_2_ to a certain gas flow rate *Q*_1_ and recorded the scattering intensity *I*_1_. Then enhanced the gas flow rate to *Q*_2_ and recorded the intensity *I*_2_, and so on to *I*_i_ and *Q*_i_.
